# Emergence of behavioural avoidance strategies of malaria vectors in areas of high LLIN coverage in Tanzania

**DOI:** 10.1038/s41598-020-71187-4

**Published:** 2020-09-03

**Authors:** K. S. Kreppel, M. Viana, B. J. Main, P. C. D. Johnson, N. J. Govella, Y. Lee, D. Maliti, F. C. Meza, G. C. Lanzaro, H. M. Ferguson

**Affiliations:** 1 Institute of Biodiversity, Animal Health and Comparative Medicine, University of Glasgow, Glasgow, UK; 2 Environmental Health and Ecological Sciences Department, Ifakara Health Institute, Dar-es-Salaam, Tanzania; 3 School of Life Sciences and Bioengineering, Nelson Mandela African Institution of Science and Technology, Arusha, Tanzania; 4 Florida Medical Entomology Laboratory, University of Florida, Vero Beach, FL 32962 USA; 5 National Vector-Borne Diseases Control Program, Environmental Health Department, P.O. Box 29, Oshakati, Namibia; 6 Vector Genetics Laboratory, Department of Pathology, Microbiology, and Immunology, School of Veterinary Medicine, University of California-Davis, Davis, CA 95616 USA

**Keywords:** Diseases, Infectious diseases, Malaria, Ecology, Behavioural ecology

## Abstract

Despite significant reductions in malaria transmission across Africa since 2000, progress is stalling. This has been attributed to the development of insecticide resistance and behavioural adaptations in malaria vectors. Whilst insecticide resistance has been widely investigated, there is poorer understanding of the emergence, dynamics and impact of mosquito behavioural adaptations. We conducted a longitudinal investigation of malaria vector host choice over 3 years and resting behaviour over 4 years following a mass long-lasting insecticidal nets (LLINs) distribution in Tanzania. By pairing observations of mosquito ecology with environmental monitoring, we quantified longitudinal shifts in host-choice and resting behaviour that are consistent with adaptation to evade LLINs. The density of *An. funestus* *s.l.*, declined significantly through time. In tandem, *An. arabiensis* and *An. funestus* *s.l.* exhibited an increased rate of outdoor relative to indoor resting; with *An. arabiensis* reducing the proportion of blood meals taken from humans in favour of cattle. By accounting for environmental variation, this study detected clear evidence of intra-specific shifts in mosquito behaviour that could be obscured in shorter-term or temporally-coarse surveys. This highlights the importance of mosquito behavioural adaptations to vector control, and the value of longer-term behavioural studies.

## Introduction

Malaria remains a major public health concern in Africa despite a vast reduction in cases and deaths over the last decade^1, 2^. Malaria parasites (*Plasmodium *sp.) are transmitted by *Anopheles* mosquitoes, with the primary vectors in Africa belonging to the *Anopheles gambiae* *s.l.* species complex and *Anopheles funestus* group^3^. Vector control, primarily using long-lasting insecticidal nets (LLINs) and indoor residual spraying (IRS), remains the primary strategy for reducing malaria transmission. Both these strategies rely on exploitation of the behavioural predisposition of many African vector species to feed on humans (anthrophagy) and rest inside houses (endophily)^4^. These interventions have generated substantial declines in malaria prevalence in many African settings^1^, including the near eradication of highly anthropophagic and endophilic vector species in some areas^5–7^.

Residual malaria transmission persists even where LLIN and IRS coverage is high^8, 9^ due to a combination of biological, social and health systems factors; with adaptive changes occurring in vector populations likely playing a major role. There has been widespread development of physiological insecticide resistance (IR) in vectors^10, 11^. Additionally, vectors may adapt their behaviour to minimize contact with insecticides in houses by, for example, biting people before they go to bed, biting and resting outdoors, or switching to feed on livestock instead of humans^12–15^. While IR has been extensively investigated and widely documented^16^, there is poorer understanding of the emergence and magnitude of behavioural avoidance strategies in malaria vectors, and their knock-on consequences for malaria control.

Behavioural avoidance could arise through different means. Firstly, interventions may trigger an ecological shift in malaria vector communities by disproportionately impacting species that are highly endophilic and anthropophagic, and skewing the composition towards species with more plastic feeding and resting behaviour^17–20^. This phenomenon is termed behavioural resilience^14, 21^. Secondly, behavioural adaptations may arise within vector species as a result of selection or phenotypic plasticity^22, 23^. Both inter- and intraspecific changes in vector behaviour pose challenges for eliminating residual malaria transmission, but evolutionary changes are particularly concerning because they may increasingly erode the effectiveness of current vector control measures and not be solvable by replacing existing insecticides with new ones.

In several areas of East Africa, shifts in vector species composition following the introduction of LLINs have been documented where *An. gambiae* declined, leaving the more behaviourally plastic *An. arabiensis* as the dominant vector (e.g.^5, 6, 24, 25^). *Anopheles arabiensis* can feed and rest outside as well as inside houses, and bite livestock and humans^26, 27^. This behavioural flexibility makes it less likely to be affected by LLINs or IRS. In contrast, *Anopheles funestus* is generally endophilic and anthropophagic^28, 29^, and has correspondingly decreased in many (e.g.^5, 6, 30^), but not all (e.g.^31^) areas after LLIN introduction. Evidence for within-species behavioural adaptations following interventions is less convincing.

Within-species changes in host choice^32, 33^, biting time and location have been reported in some settings^13, 34^. Several studies have identified a genetic basis for these behaviours (e.g.^23, 35^), indicating their potential to respond to selection. However, estimation of the rate, magnitude and implications of mosquito behavioural adaptations has been limited by lack of systematic long-term data and inconsistencies in methodologies. For example, several studies of mosquito behaviour in response to control are based on short-term “before” vs “after” comparisons^36–38^; often using historical data collected using different methods by different teams at different times^25, 39^. Furthermore, comparisons of malaria vector behaviour are often made across periods where environmental conditions as well as vector control pressure have changed, making it difficult to disentangle their respective impacts. To address these gaps, here we conducted fine-scale longitudinal sampling of the host choice and resting behaviour of the malaria vectors *An. arabiensis* and *An. funestus* *s.l*. at several sites over a 4-year period following a mass LLIN distribution campaign in Southern Tanzania^40, 41^. Our aims were to test for temporal changes in mosquito vector abundance, resting habitat (in versus outdoors) and host choice (human versus livestock) consistent with the emergence of behavioural avoidance strategies. By sampling at multiple sites over seasonally-varying conditions, longer-term trends in behavioural phenotypes were disentangled from environmental variation.

## Results

In a longitudinal study, data on the indoor and outdoor resting and indoor host-seeking malaria vectors *Anopheles arabiensis* and *Anopheles funestus *s.l. was collected in 4 villages over the course of 4 years. Resting mosquitoes were captured using backpack aspirators indoors and in animal sheds, and resting bucket traps outdoors, while host-seeking mosquitoes were trapped with CDC light traps indoors. We tested the effect of environmental (season and saturation deficit derived from temperature and relative humidity) and household variables (livestock presence, distance to breeding sites, number of nets present, and house type) on several measures of abundance and host-choice over time. Saturation deficit, derived from temperature and humidity measurements was used. It is the deficit between the amount of moisture in the air and the amount of moisture the air can hold when it is saturated, making it a more meaningful measure for micro-climatic effects on insects which try to avoid desiccation ^42, 43^. Host-choice of *An. arabiensis* was investigated by analysing the proportion of mosquitoes who fed on humans out of the total of blood-fed mosquitoes tested. Results presented below describe the predicted impacts of variables that had a significant association with entomological parameters of interest.

### Mosquito vector abundance

There was an appearance of decline in the abundance of indoor host-seeking *An. arabiensis* across the study period, albeit not significant. The abundance of *An. arabiensis* varied significantly with saturation deficit and household livestock presence (Table 1, Supplementary Material 1); being greater at a lower saturation deficit, and at households without livestock (Table 1, Supplementary Material 2). None of the other household level variables (distance to breeding site, number of nets, house type) were significantly related to *An. arabiensis* host-seeking abundance.

**Table 1:** The predicted mean abundance of female *An. arabiensis* and *An. funestus *s.l. caught by different trapping methods: (CDC light traps indoors, resting collections inside houses, animal sheds and outdoors) and their Human Blood Index (HBI, proportion of identified blood meals taken from humans).

Trait	Vector species	Predicted mean estimate (95% CI)						
		Start of study	End of study	Wet season	Dry season	HH with livestock	HH without livestock	Mean abundance decrease per unit increase in saturation deficit
Abundance host seeking indoors	*An. arabiensis*	52.69 (48.68–57.03)	39.8 (13.14–120.56)	10.33 (6.9–15.5)	6.88 (4.04–11.7)	5.9* (3.7–9.5)	13.9* (9.2–20.9)	9.1* (6.3- 13.1)
	*An. funestus* *s.l.*	29.6* (27.3–32)	2.94* (1–9)	4.2 (2.04–8.66)	6.77 (3.05–15.00)	4.57 (2.07–10.1)	5.04 (2.39–10.6)	4.85* (2.4 -9.95)
Abundance resting inside houses	*An. arabiensis*	0.94* (0.88–1)	0.17* (0.07–0.44)	0.34 (0.25–0.46)	0.48 (0.32–0.72)	0.46 (0.32–0.65)	0.34 (0.25–0.46)	0.38 (0.29–0.49)
	*An. funestus* *s.l*.	3.1* (2.9–3.3)	1.38* (0.6–3.2)	0.62* (0.37–1.04)	1.07* (0.68–1.83)	1.04* (0.6–1.8)	0.64* (0.38–1.08)	0.81* (0.49–1.35)
Abundance resting outside houses	*An. arabiensis*	0.62 (0.59–0.65)	0.32 (0.15–0.67)	0.89 (0.63–1.26)	1.08 (0.72–1.63)	1.25* (0.88–1.77)	0.72* (0.5–1)	0.9 * (0.69–1.31)
	*An. funestus s.l.*	0.25 (0.24–0.26)	0.16 (0.08–0.32)	0.14* (0.01–0.19)	0.28* (0.2–0.4)	0.26* (0.19–0.37)	0.15* (0.11–0.21)	0.2* (0.15–0.27)
Abundance resting in animal sheds	*An. arabiensis*	3.97* (3.45–4.57)	0.51* (0.07–3.72)	0.17 (0.08–0.33)	0.49 (0.17–1.40)	NA	NA	0.22* (0.12–0.39)
Human Blood Index overall	*An. arabiensis*	0.21* (0.18–0.23)	0.07* (0.002–0.2)	0.03* (0.02–0.06)	0.13* (0.07–0.25)	0.03* (0.02–0.05)	0.16* (0.1–0.27)	NA
Human Blood Index indoors	*An. arabiensis*	0.85 (0.81–0.88)	0.48 (0.09–0.89)	0.06* (0.03–0.15)	0.36* (0.12–0.7)	0.05* (0.017–0.16)	0.41* (0.22–0.62)	NA
Human Blood Index outdoors	*An. arabiensis*	0.49 (0.44–0.54)	0.25 (0.07–0.6)	0.05* (0.03–0.09)	0.18* (0.1–0.32)	0.05* (0.02–0.1)	0.2* (0.12–0. 3)	NA
Human Blood Index Animal Shed	*An. arabiensis*	0.0006 (3.24e−04–0.001)	0.005 (3.4e−05–0.44)	0.02 (6.39e−04–0.33)	0.002 (3.23e−05–0.07)	NA	NA	NA

Mean values for abundance are those obtained from the best model with all other variables held constant. Values in brackets are 95% confidence intervals. Values for the “start of study” are the predicted means for the 1st 3-month block of the study (Jan–Mar 2012), and “end of study” refers to the last 3-month block. The “end of study block” was March–May 2015 for abundance data, and April–June 2014 for Human Blood Index. Mean abundance decrease per unit increase in saturation deficit was calculated with all other variables held constant. Asterisks denote significance of the variable (p < 0.05). “NA” denotes not applicable for the variable tested. “HH” denotes household.

**Figure 1:**
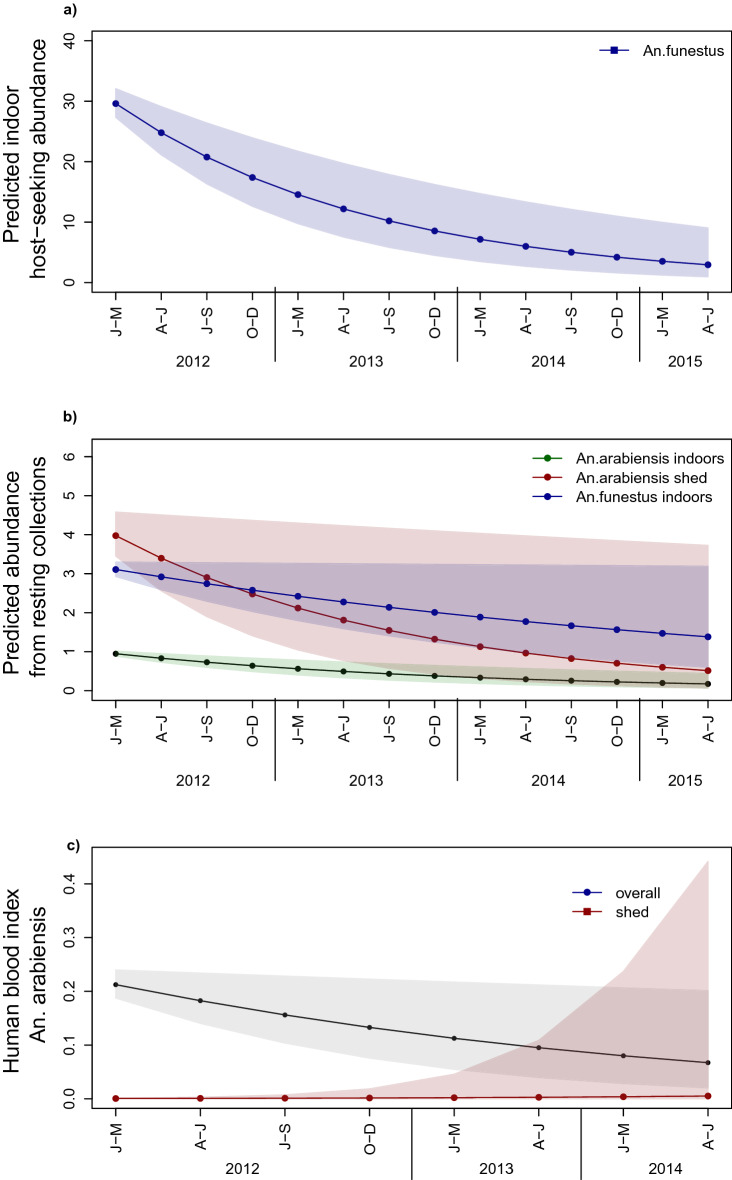
Predicted mean mosquito abundance per trap per night with 95% confidence interval (**a**) host-seeking *An. funestus s.s.* from January 2012 to May 2015 indoors (**b**) resting *An. arabiensis* and *An. funestus s.s*. indoors and *An. arabiensis* in animal sheds from January 2012 to May 2015 and (**c**) Human blood index of *An. arabiensis* overall and in animal sheds from January 2012 to June 2014. Non-significant effects were not retained in the best model; therefore, predictions for these were not available. Raw data is shown in Supplementary Figs. S1 and S2.

The abundance of host seeking *An. funestus s.l.* declined tenfold between the start and end of the study (Table 1, Fig. 1a, Supplementary Material 2). Unlike *An. arabiensis*, there was no significant impact of household livestock presence on the abundance of host seeking *An. funestus s.l.*, but a higher saturation deficit (higher dryness) negatively impacted mosquito numbers (Table 1). None of the other household-level variables was significantly related to *An. funestus s.l.* host seeking abundance.

### Resting behaviour of malaria vectors

The number of *An. arabiensis* found resting inside houses consistently declined over the 4-years of study (Fig. 1b, Supplementary Material 3 and 4); falling from ~ 1 resting mosquito per house per night in the first 3 months of the study to 0.17 by the end (Table 1). In contrast, the number of *An. arabiensis* resting outdoors was highly variable and showed no consistent change over time (Table 1, Supplementary Material 5). More *An. arabiensis* were found resting outdoors at households with livestock. As saturation deficit increased (the air became drier), fewer *An. arabiensis* were found resting outdoors and in animal sheds (Table 1, Supplementary Material 4 and 5). Significantly more *An. arabiensis* rested inside animal sheds than inside houses or outdoor resting boxes (Table 1, Supplementary Fig. S1), but the mean number of mosquitoes in animal sheds declined more than sevenfold over the study period (Table 1, Fig. 1b, Supplementary Materials 4, 6). The longitudinal decline in the number of *An. arabiensis* resting inside houses and animal sheds, while densities outdoors remained relatively constant indicates there was an overall increase in exophily across the study period.

Similar to *An. arabiensis,* the decline of the mean number of *An. funestus s*.*l.* found resting indoors was significant over the 4-year study period, if marginally (Table 1, Fig. 1b, Supplementary Materials 3, 4). In addition, the abundance of *An. funestus s.l.* in indoor resting collections was higher in the dry than wet season and at households with than without livestock (Table 1). The number of *An. funestus s.l*. resting indoors declined as saturation deficit increased (Table 1). Similar to *An. arabiensis*, the number of *An. funestus s.l.* resting outdoors did not vary significantly over the study period (Table 1, Supplementary Fig. S1). Twice as many *An. funestus s.l*. were found in outdoor resting collections in the dry than wet season, more at households with than without livestock, and as saturation deficit decreased (Table 1, Supplementary Materials 4, 5). Too few *An. funestus s.l.* were found resting inside animal sheds (n = 112) to undertake robust analysis of longitudinal trends. As with *An. arabiensis,* the combination of a consistent longitudinal decline in the indoor but not outdoor resting density of *An. funestus s.l.* indicates there was a significant shift towards exophily over the study period (Table 1).

### Changes over time in malaria vector host-choice

Not enough blood fed *An. funestus s.l.* were collected to validate blood meal analysis. All of the 2,152 blood fed *An. arabiensis* captured between 2012 and 2014 underwent blood meal analysis. Ninety-nine percent (n = 2,140) had fed on one or a mixture of hosts (human, cattle, goat, pig, sheep, dog or chickens), with the remainder being unidentified. The overall HBI in *An. arabiensis* was significantly associated with time period, season, trapping method and livestock presence (Table 1, Supplementary Materials 7, 8). The proportion of blood meals that *An. arabiensis* took from humans was predicted to decline from 21 to 7% over the 3-year study period (Table 1, Fig. 1c); and was significantly higher in resting indoors (50%) than outdoors (24.1%) or in animal sheds (9.1%, Table 1). Additionally, the HBI of *An. arabiensis* was significantly lower at households where livestock were present and during the wet season. This decline was most evident in *An. arabiensis* caught resting outdoors (Table 1). The HBI of *An. arabiensis* caught resting indoors was also significantly lower at households with livestock and during the wet season than the dry season (Table 1).

### Changes over time in environmental variables

In tandem with mosquito collections, we tested for longitudinal variation in microclimatic conditions, presence of livestock, house construction (e.g. % mud walls), distance to closest breeding site and number of bed nets reported at each surveyed household (Supplementary Material 9); with the aim of identifying environmental factors that may vary with mosquito abundance and behaviour. Of these, only the proportion of livestock ownership increased in the surveyed households over time (coef = 16.8, p value < 0.001; Supplementary Material 9b). There was no significant inter-annual change in house type, mean distance to breeding sites, number of nets per household, mean temperature, humidity and saturation deficit (Supplementary Material 9b). The CHIRPS rainfall dataset did not show any anomalies in rainfall for the study period; with rainfall patterns staying much the same across years and no significant rise or fall (Supplementary Material 9c).

## Discussion

This study demonstrated a systematic temporal shift in two epidemiologically-relevant mosquito behavioural traits over the 4 years following a mass LLIN distribution in Tanzania. These behavioural shifts coincided with a decline in *An. funestus s.l*. density over the study period, suggesting they may reflect adaptations in response to selection imposed by LLINs. Previous studies have documented changes in behaviors at the mosquito species complex-level following interventions^18, 19, 28^, but lacked resolution to distinguish changes occurring within species from ecological shifts in species composition. Consistent with the hypothesis of behavioral changes arising as an adaptive strategy, this study unambiguously identified phenotypic shifts within *An. arabiensis.* Behavioural shifts were also detected within *An. funestus s.l.*; which was assumed to be mainly *An. funestus s*.*s*., based on a concurrent study of Lwetoijera *et al*.^44^. Both vectors became increasingly exophilic over the study period, with *An. arabiensis* also increasingly shifting its host choice from humans to cattle, while too few *An. funestus s.s*. were caught for host-choice analysis. The direction of these shifts is consistent with the development of behavioral avoidance strategies to avoid contact with indoor-based interventions.

Although there was evidence of behavioural change in both mosquito vectors studied here, its range and magnitude varied. While the estimates derived from the different trapping methods for resting mosquitoes may not be suitable for quantifying the absolute degree of exophily, as this would require calibration of each method against an unbiased estimator of population density, it can infer a relative trend. We did not detect a temporal reduction outdoors for *An. fu*nestus *s.s*., which could be due to the relatively small sample size (n = 155) and the resulting insufficient statistical power to detect a change.

As expected from their previously described ecology (e.g.^44–46^), *An. funestus s.s*. was more likely to rest indoors compared to the more zoophilic and exophilic *An. arabiensis.* While the density of both vectors fell over time, the decline was significant only, and much more pronounced in *An. funestus s.s*. This observation is consistent with the prediction that LLIN should be most effective against endophilic species, as has been observed in other studies in west (e.g.^47^) and east Africa (e.g.^5, 25^)*.* Due to the lack of non-intervention “control” areas, we cannot exclude the possibilities that this longer-term decline in vector density could be due to other types of concurrent environmental change. However, we did not detect any systematic changes in key environmental (temperature, rainfall, distance to breeding sites) or housing factors (wall type and LLIN number) that impact vector densities across years. Thus we hypothesize that pressure from LLINs is the most likely explanation for continued fall of *An. funestus s.s*. densities and a shift to greater exophily for both vectors over the study period.

In addition to longitudinal declines over the study period, malaria vector abundance also varied with saturation deficit—a measure for the drying power of air, derived from air pressure, temperature and relative humidity. The density of host seeking vectors was significantly higher in moister conditions (low saturation deficit) as expected (e.g.^48^). For host-seeking vectors, there was no significant seasonal variation in abundance. Overall mean indoor host-seeking vector abundance in the area over the study period from 2012 to 2015 was similar for both vectors to collections by Lwetoijera *et al.*^44^ from 2008 to 2012 and Mayagaya *et al*. from 2007 to 2009^45^. A marked decline in the *An. gambiae s.l*. complex can be seen in the study area between CDC catches from 1990 to 1994 reported by Russell et al.^49^ and later studies from 2007 to 2009 and 2008 to 2012 by Mayagaya *et al.* and Lwetoijera *et al*.^44^, respectively, and finally from this study. This reflects evidence of a much longer-term decline in indoor abundance throughout the area together with intensive LLIN distribution.

In outdoor resting collections, *An. funestus s.s*. numbers were higher in relatively dry than wet months. In the same area, *An. funestus s.s*. has previously been found to be similarly abundant in the wet and dry season (e.g.^44^), with abundance recently positively correlated with rainfall with two months’ lag previously^50^. No seasonal variation in the abundance of resting *An. arabiensis* was evident. Major environmental determinants of vector abundance and behavior were household livestock ownership and saturation deficit. The abundance of *An. funestus s.s*. resting in- and outdoors, and of *An. arabiensis* resting outdoors and in animal sheds, decreased with increasing saturation deficit (e.g. as air became drier). This is consistent with an increased risk of desiccation-related mortality^51^.

The impacts of livestock were widespread; boosting the abundance of outdoor resting *An. arabiensis,* and indoor and outdoor resting *An. funestus s.s*. Immigration of zoophilic *Anopheles* populations, genetically different to the historic population in the area, is possible as a genetic background associated to host choice in *An. arabiensis* from the same collections has been found^23^. This has implications for possible vector control methods. Both *An. arabiensis* and *An. funestus* were found outdoors, with the former increasingly feeding on cattle through time, reinforcing the potential value of complementary zooprophylaxis strategies. In particular, strategies that extend coverage of interventions to cattle, such as insecticide treatments^52^, use of endectocide like ivermectin^53^ could have particular value for crashing *An. arabiensis* populations.

The *An. funestus s.l.* specimens were not identified to species level in our study, therefore it is possible that they could belong to several cryptic species and that certain changes detected were species composition changes and not within-species changes. Other studies in the same area (e.g.^31, 54^) found that the predominant species in the area is *An. funestus s.s*., however, Meza et al. found that ~ 30% outdoor caught *An. funestus s.l*. were *An. rivolurum* and *An. leesoni*.

The Human Blood Index of *An. arabiensis* was substantially lower at households where livestock were present, consistent with other studies showing a zooprophylactic effect of livestock found in this^46^ and other settings^55, 56^. It is notable that both vectors, even the more anthrophilic *An. funestus s.s*., were more abundant in outdoor resting sites in the presence of livestock. In the study area, while Meza *et al*. found that ~ 30% outdoor caught *An. funestus s.l.* were *An. rivolurum* and *An. leesoni*, the predominant species remains *An. funestus s.s*. as also found by Kaindoa *et al*. In Meza’s study about 75% of *An. funestus s.s*. attempted to feed on humans, confirming its anthropophily^31, 54^^.^*.* While too few blood fed *An. funestus s.s*. were collected for analysis here, a previous study in this area found that the HBI of this species fell from ~ 100 to 50% when livestock were present at households^45^. Thus we hypothesize that increased exophily in *An. funestus s.s*. in the presence of livestock is because they are diverted from feeding on people by livestock. A systematic review reported that cattle provide a zooprophylactic effect only in cases when the dominant mosquito vector species prefer livestock to humans^57^; however, our results suggest this effect may arise even in vectors that are relatively anthrophilic like *An. funestus s.s*. here. The ubiquity of livestock presence as a predictor of malaria vector abundance, resting behaviour and host choice here highlights the strategic value of extending vector control measures to cover cattle in this and other African settings (e.g.^52, 58^).

These findings have implications for current and future vector control strategies. First, the continuing reduction in malaria vector density of *An. funestus s.s*., over a 4-year period following an LLIN distribution, indicates a sustained impact of the intervention, with no evidence of a rebound in vector density. We also detected a significant increase in exophily in both *An. arabiensis* and *An. funestus s.s*., and a substantial decrease in the HBI of *An. arabiensis*. We hypothesize that these behavioural shifts reflect adaptations to minimize contact with LLINs. An association between human host choice and chromosomal inversion has been identified in these *An. arabiensis* populations (e.g.^23^); indicating this phenotype has a genetic basis and could respond to long-term selection from interventions that impose a fitness cost on human-feeding (e.g. LLINs). No similar association between resting behavior and chromosomal inversions was detected in this *An. arabiensis* population^23^; but have been found in other African *Anopheles* populations (e.g.^59^). Further investigation is required to confirm the genetic basis of these mosquito behaviour patterns and their potential to respond to selection. However, the systematic long-term shifts in these traits here against a backdrop of population decline are consistent with the emergence of behavioural avoidance strategies in response to LLINs.

The relative importance of behavioural avoidance in vectors to ongoing malaria transmission remains unclear, emphasizing the need for further exploration^60^. Both mosquito behaviours studied here, resting location and host choice, showed evidence of temporal shifts. Other mosquito behaviour of crucial epidemiological importance include the time and place of biting (in or outside, before or during sleeping hours) were not measured here. However, a recent modelling investigation of data from across Africa indicates there is evidence of a weak, but statistically significant decline in the percentage of bites taken by malaria vectors when people are protected by LLINs (e.g. when indoors and asleep), of sufficient magnitude to cause ~ 10 million malaria cases^9^. In combination with the shifts in resting and host choice described here, similar shifts in malaria vector biting behaviour could pose complex and diverse challenges to vector control. Insecticide resistance is generally viewed as the most epidemiologically important mosquito adaptation to control. While insecticide resistance was not measured in this study, concurrent studies in the study area confirmed insecticide resistance in *An. arabiensis* and *An. funestus s.l*.^44, 61^. The behavioural shifts in vector populations described here could either mitigate or enhance the impacts of insecticide resistance. For example, the shift to outdoor resting and zoophily would be expected to reduce contact with insecticides indoors, and thus possibly selection for physiological resistance. The simultaneous emergence of both physiological and behavioural resistance strategies could erode the impact of indoor-based insecticide control strategies more than any one on their own. Understanding the interplay of mosquito physiological and behavioural adaptations to insecticides will be vital to predicting the sustainability of indoor-based interventions^60^. The clear evidence of within-vector behavioural shifts presented here confirms the urgent and growing need for new control strategies, including those that target vectors outside of houses and/or feed on cattle^58^ and highlights the value of incorporating routine surveillance of vector behaviour into malaria control programmes.

## Materials and methods

### Study area

**Figure 2:**
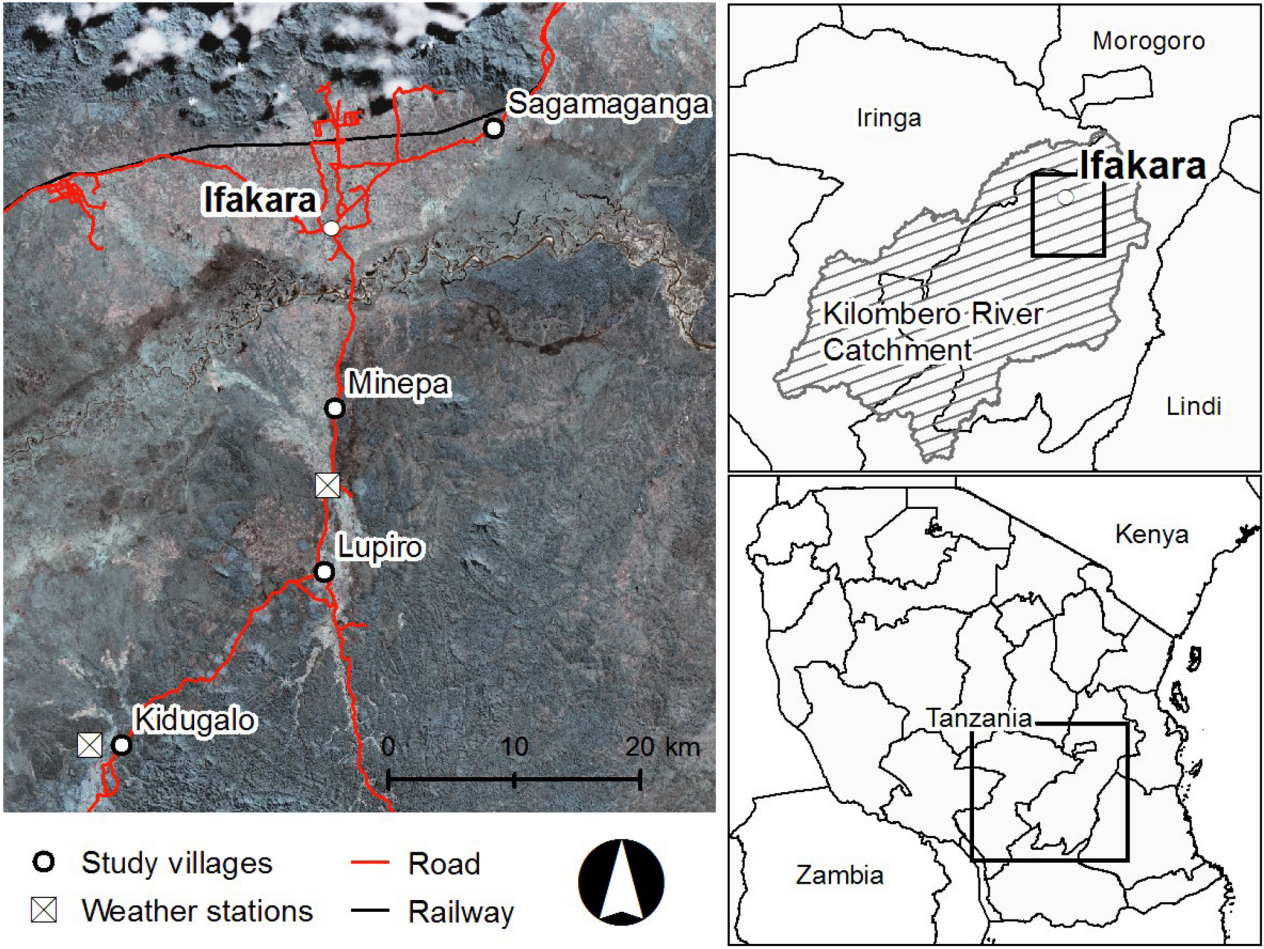
Study site in the Kilombero Valley in Kilombero and Ulanga districts, Tanzania, showing Ifakara and the four study villages as well as the weather stations. Entomological and environmental data was collected for all four villages. (Generated by ArcGIS 10.2, https://www.esri.com/software/arcgis/arcgis-for-desktop).

This study was conducted from January 2012 to May 2015 in the Kilombero River Valley of southern Tanzania (7° 44′ to 9° 26° S/35° 33′ to 36° 56′ E). After the scaling up of insecticide-treated bed nets (ITNs) from 2004 and LLINs from 2009 to 2011^40, 41^, a decline in malaria vector numbers and malaria transmission was seen in Tanzania. A National Voucher Scheme provided a voucher to pregnant women and infants when visiting a reproductive and child health (RCH) facility greatly reducing the price for ITNs and LLINs. Between 2008 and 2010, the “Under 5 Catch–Up Campaign” distributed LLINs countrywide to all children under 5 years of age. Additionally, in January 2011, a year before the study started, a universal coverage campaign led to a further mass-distribution of LLINs over 3 days in each town and village in the study area. As a result, ownership of at least 1 ITN per household increased from ~ 45% in 2008 to 91.5% in 2011, in Tanzania, including the Kilombero Valley^40^, with a mean number of 2.5 nets per household^41^. Malaria vectors were collected from 4 villages: Kidugalo (KID), Lupiro (LUP), Minepa (MIN) and Sagamaganga (SAG, Fig. 2). Lupiro and Minepa are inhabited predominantly by rice farmers and are surrounded by paddies, while both rice farming and livestock keeping are common in Kidugalo and Sagamaganga. The primary malaria vectors in this area are *Anopheles arabiensis* and the *An. funestus *sensu lato (*s.l*.) species complex^62^, which, although not identified to species level here, was likely to be mainly composed of *An. funestus* *s.s*., based on concurrent studies of Lwetoijera et al. and Meza et al.^44, 54^. Malaria vector species composition and abundance have been extensively described^45^.

### Experimental design

Vector surveillance was carried out to identify long-term shifts in the ecology (abundance and species composition) and behaviour (resting habitat and host choice) of malaria vectors. After a mass-distribution of LLINs (Olyset Net LLIN with permethrin) from 2008 to 2011, mosquitoes were sampled in 4 villages over 10 periods between 2012 and 2015 (Supplementary Material 10a). This generated mosquito collections from 350 households over 199 nights. On the first day of each sampling block, an index house was selected on the basis of being accessible and the presence of residents to participate. Additional houses were recruited in the vicinity of the index house to achieve the required sample size (4–10 households per village), with spacing between individual houses not more than 100–200 m.

### Trapping methodology

Each sampling day, mosquitoes were collected using three methods: (1) CDC light traps (CDC, Model 512, John Hock, Gainesville, FL, USA) placed indoors to provide a proxy of overall mosquito abundance and human biting rate^63^. These traps were placed ~ 1.5 m above the foot end of a bed occupied by people sleeping under an LLIN, and ran between 18:00 and 6:00. (2) CDC backpack aspirator (BP, Model 1412, John Hock) were used to collect mosquitoes resting inside houses and animal sheds (at households with livestock). The nozzle of the BP was swept over the interior walls and ceiling for ten minutes^64^ in another room from CDC light traps to reduce trap interference. Roofs and walls of sheds or paddocks of animal enclosures were aspirated at houses with livestock. (3) Resting buckets (RBu)^65^ were used to sample mosquitoes resting outdoors in the peri-domestic area. Seven RBu traps were placed outside (2–10 m from house) in the first 3 sampling rounds and 10 RBu per household thereafter, to increase the number of mosquitoes caught. This change was controlled for in the analysis. Mosquitoes resting inside RBus were collected at dawn using a BP aspirator. All resting collections were made between 6:00 and 8:00.

### Environmental data

Global positioning system (GPS) coordinates were recorded for each household. Data loggers (Tiny Tag plus 2; Gemini data loggers, UK, Ltd) recorded temperature and humidity inside houses at ~ 1 m above ground. This was used as measurement of overall temperature and humidity, based on the assumption that general seasonal trends follow a similar pattern in and outdoors. Saturation deficit, indicative of general “drying power of the air” was calculated following Allen et al.^66^, even though for simplification this was only estimated using indoor microclimatic data here.

Monthly rainfall data from the Climate Hazards group Infrared Precipitation with Stations (CHIRPS) dataset at 0.05° × 0.05° spatial resolution and data from 2 local weather stations (Fig. 2) was used for the study period^67^. The “season” of collections was defined as wet or dry based on the region’s rainfall data (Supplementary Material 10b). At all households, before setting the traps, we recorded the presence of livestock (defined as cattle, goat, pig, or sheep), the number of LLINs being used and housing type (brick or mud-walled). The horizontal distance to the nearest *Anopheles* mosquito breeding site (water bodies likely to harbour *Anopheline* larvae such as rice paddies, edges of streams, water ditches and ponds) was estimated with 100 m accuracy by pacing in a straight line.

### Mosquito identification and molecular analyses

Trapped mosquitoes were killed by chloroform and number and sex of those morphologically identified as belonging to the *An. gambiae s.l.* complex or *An. funestus s.l*. group recorded. A subset of *An. gambiae s.l.* collected in CDC light traps (n = 1692, 6.7% of total) were identified to species level by PCR^68^, with all confirmed as being *An. arabiensis*. All *An. gambiae s.l*. collected are thus assumed, and hereafter defined, as *An. arabiensis* due to their predominance in our samples and other studies in the area^69^. *Anopheles funestus* *s.l.* specimens were not identified to species level, but as stated above, believed to be primarily *An. funestus* *s.s*. from concurrent studies in the area^44^.

Of the blood-fed *An. arabiensis* from resting collections, 14% were from animal sheds, 23.5% from indoor aspirations and 62.5% from outdoor RBUs (n = 2,131). All were identified to species level (16.3% of total collected) and had their blood meal identified via PCR. Cytochrome b sequences from 6 host species were collected from Genbank and consensus sequences were generated. SNPs informative for each host were then selected for genotyping. This was performed by extracting DNA^23^ and using a multiplex genotyping assay to distinguish between cattle, goat, pig, dog, chicken and human blood^70^.

Sample sizes used in the analysis for each dataset are provided in Supplementary Material 11. Genetic information and Meta-data associated with this study are available in the PopI database: AaGenome (https://popi.ucdavis.edu/PopulationData/OpenProjects/AaGenome/).

### Statistical analysis

Temporal changes in mosquito abundance, species composition, resting behavior and host choice were investigated using generalized linear mixed models (GLMMs). Mosquito abundance was estimated as the mean number caught per CDC light trap and per indoor aspiration and outdoor RBU resting collection per night respectively. Changes in the mean abundance of malaria vectors resting in different habitats (inside houses, animal sheds and outdoors) were estimated to assess shifts in resting behavior. Changes in the trapping effort of resting mosquitoes outside (number of RBUs per house) were controlled for by including the number of buckets per household as an offset in the model. Correlation between indoor and outdoor resting mosquito was not tested for, because of the different trapping methods with different efficacies used. Direct comparison of these methods is not straightforward and the proportion or correlation between them difficult to interpret. Host choice for *An. arabiensis* only, was measured in terms of the Human Blood Index (HBI, proportion of human blood fed females out of the total identified). Sample sizes in all analyses are provided in Supplementary Material 11. Temporal changes were investigated by modelling time as a continuous variable from the start to end of the study. The entire study period (January 2012–May 2015) was divided into units of 3 months (timepoints) so that each “time period” encompassed one round of data from all 4 villages. Mosquito count data was modelled using either a Poisson or Negative Binomial depending on the degree of overdispersion (following Cameron et al*.*^71^).

Variation in mosquito abundance was modelled separately for indoor host-seeking, indoor resting, and outdoor resting as a function of time, season and other environmental variables and household characteristics (Supplementary Material 1). Sampling day, household ID and village were included as non-nested random effects. Analysis of temporal changes in the HBI in *An. arabiensis* was based on data from only three study sites as too few blood-fed mosquitoes were collected in Kidugalo for robust analysis. Four separate GLMMs were constructed to test for temporal changes in the HBI of *An. arabiensis* caught overall, resting inside houses, animal sheds and outdoors respectively. Here, HBI was modelled as a binomial variable with blood meals with any traces of human blood labelled as positive and all others as negative. We tested for a linear association with time, season (wet/dry) and livestock presence at the household (Supplementary Material 9y). Date, household ID and village were included as random effects.

For all response variables, model selection was based on backward elimination from an initial maximal model that included all fixed and random effects of interest (Supplementary Materials 1, 3, 5, 6, 7). The significance of individual variables was tested using likelihood ratio tests. All statistical analyses were carried out using the glmmTMB and lme4 packages in R 3.5.2.y^72–74^.

## Supplementary information

Supplementary Figure 1

Supplementary Figure 2

Supplementary Material 1

Supplementary Figure Legends
