# Supplementary material for: Emergence of behavioural avoidance strategies of malaria vectors in areas of high LLIN coverage in Tanzania
Source: Sci Rep. 2020 Sep 3;10:14527. doi: 10.1038/s41598-020-71187-4 (PMC7471940; doi:10.1038/s41598-020-71187-4)
Supplement: Supplementary file 1 — Supplementary Figure 1 [file 41598_2020_71187_MOESM1_ESM.pdf]

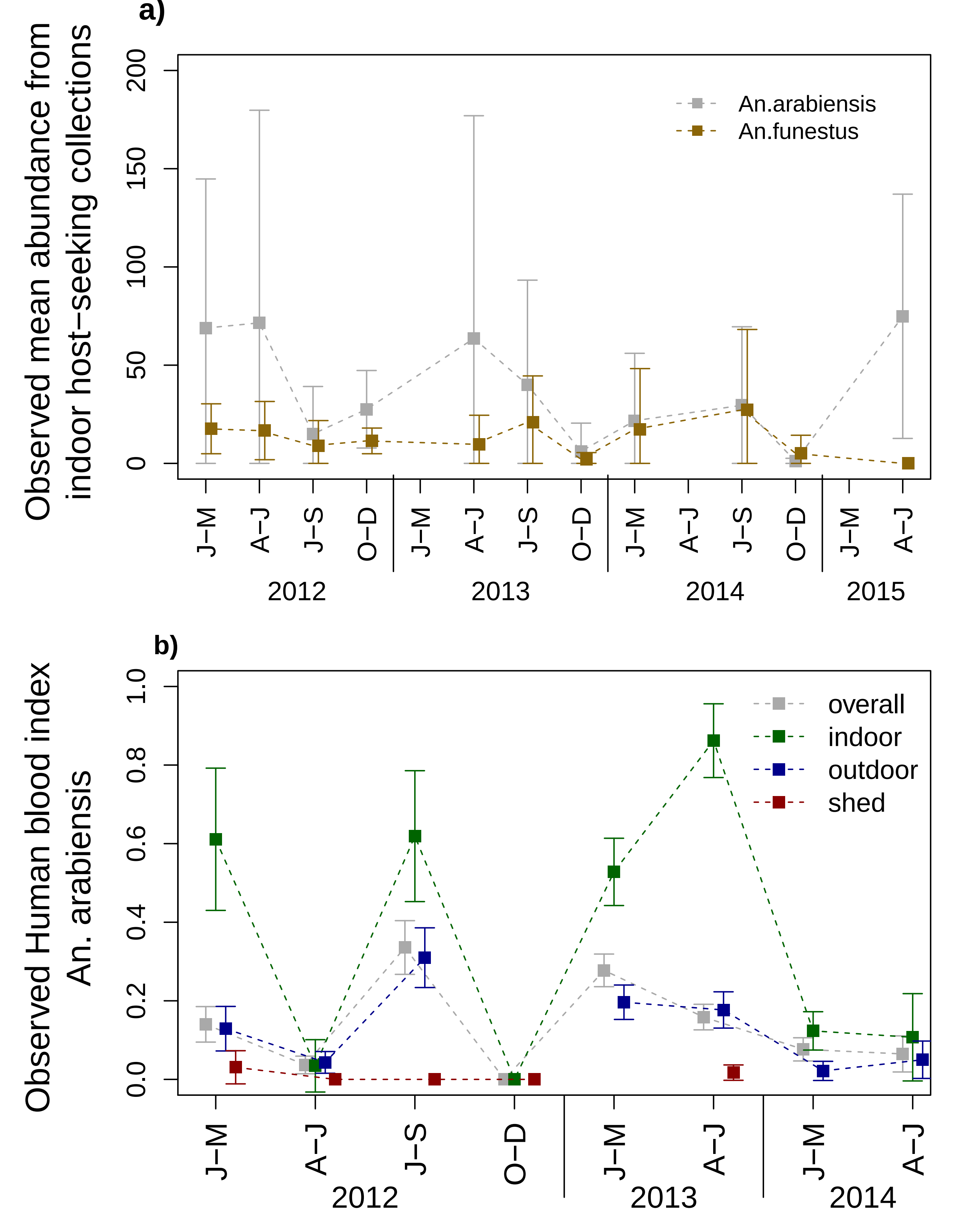
a)
Observed mean abundance from indoor host−seeking collections
0
50
100
150
200
An.arabiensis
An.funestus
J−M
A−J
J−S
O−D
2012
2013
2014
2015
b)
Observed Human blood index An. arabiensis
0.0
0.2
0.4
0.6
0.8
1.0
overall
indoor
outdoor
shed
J−M
A−J
J−S
O−D
2012
2013
2014
