# Supplementary figures and images for: Emergence of behavioural avoidance strategies of malaria vectors in areas of high LLIN coverage in Tanzania
Source: Sci Rep. 2020 Sep 3;10:14527. doi: 10.1038/s41598-020-71187-4 (PMC7471940; doi:10.1038/s41598-020-71187-4)

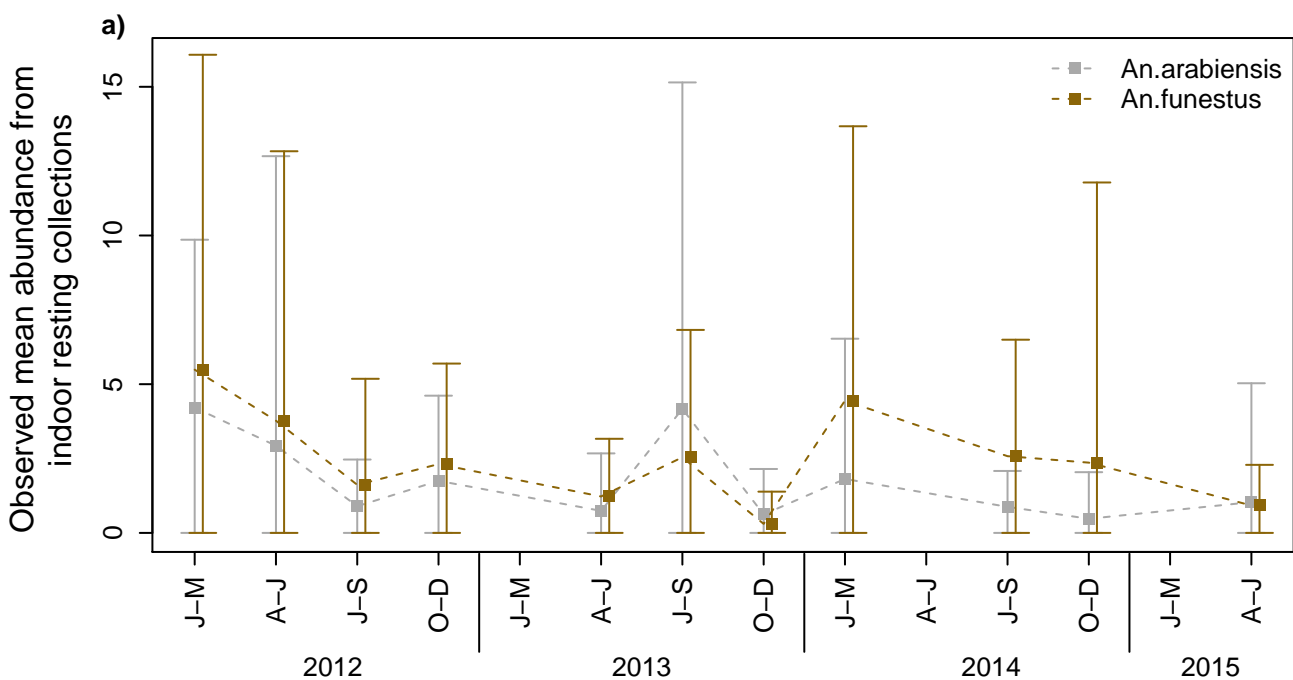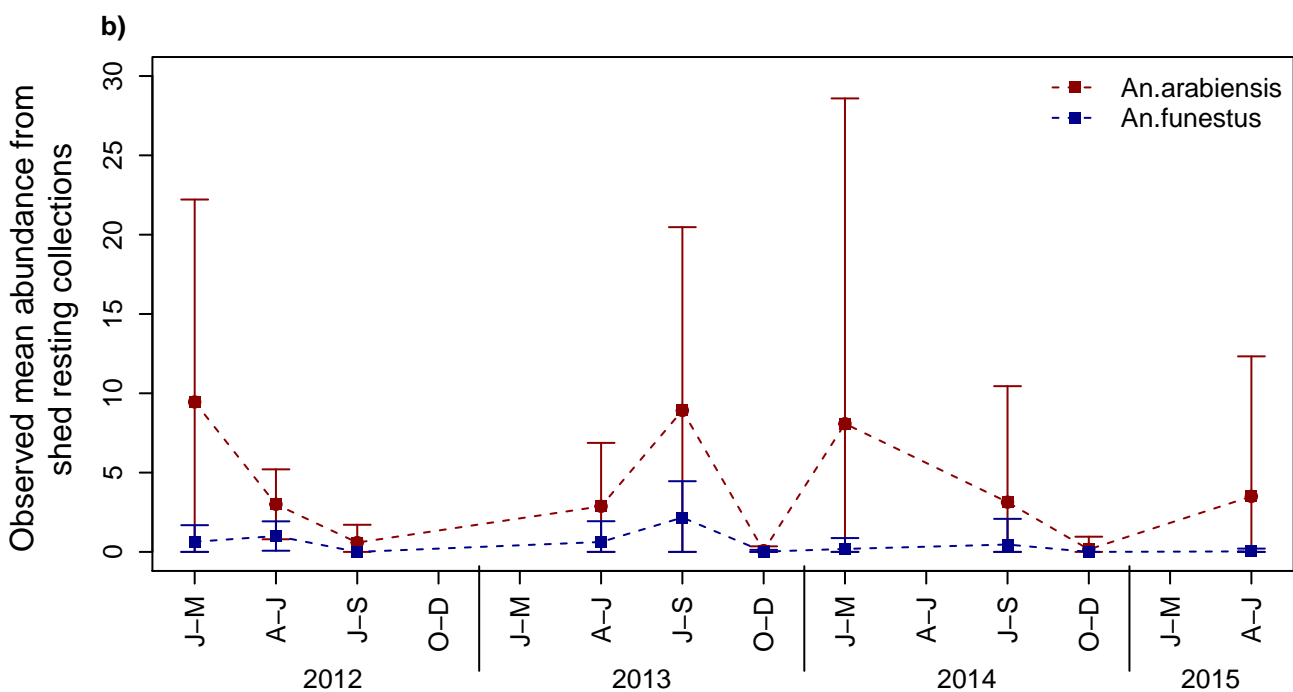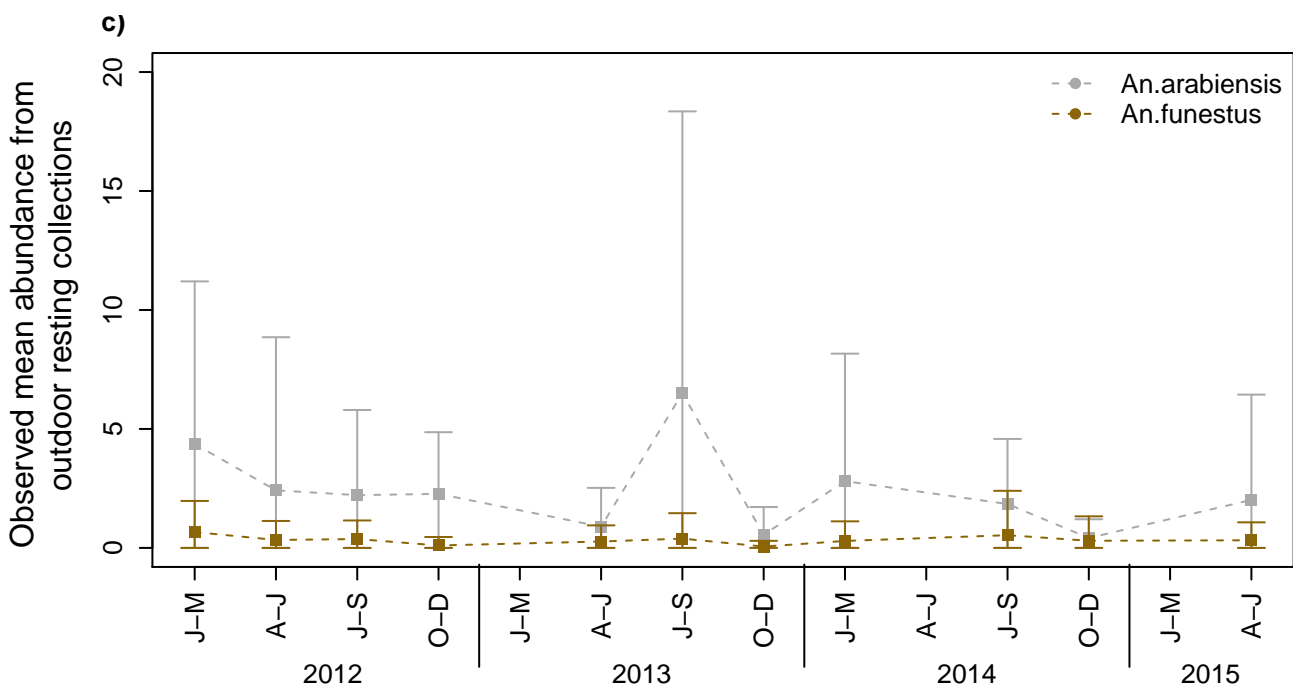

Supplement: Supplementary file 2 — Supplementary Figure 2 [file 41598_2020_71187_MOESM2_ESM.pdf]
