# Supplementary material for: Emergence of behavioural avoidance strategies of malaria vectors in areas of high LLIN coverage in Tanzania
Source: Sci Rep. 2020 Sep 3;10:14527. doi: 10.1038/s41598-020-71187-4 (PMC7471940; doi:10.1038/s41598-020-71187-4)
Supplement: Supplementary file 3 — Supplementary Material 1 [file 41598_2020_71187_MOESM3_ESM.docx]

**Supplementary Materials for:**

**Emergence of behavioural avoidance strategies of malaria vectors in areas of high LLIN coverage in Tanzania.**

K. S. Kreppel ^1, 2, 3^ *, M. Viana ^1­­^, B. J. Main ^4^, P. C. D. Johnson ^1^ , N. J. Govella ^2^ , L. Yoosook, D. Maliti ^5^, F. C. Meza ^2^ , G. C. Lanzaro^4^, H. M. Ferguson ^1^

**Supplementary Material 1:** Model selection for indoor host seeking *An. arabiensis* and *An. funestus* *s.s.* with the chosen model marked in bold. Models were selected on the basis of significance of variables to the model fit, AIC-values and rule of parsimony as well as visual inspection of the plotted residuals against the fitted values. Time = Time point 1 to 14; Season = Wet/Dry; Livestock = Livestock owned (Yes/No); Sat. Deficit = Saturation Deficit of the air measured indoors; Distance Water = Estimated distance to closest breeding site; Nets = Number of LLINs used in household; House type = Building material house is mainly made of (mud/brick); Village = Village name (KID, LUP, MIN, SAG); Household = House ID; Date = Day/Month/Year of data collection.

| **Variable of interest** | **Name** | **Species** | **Fixed Effects** | **Random Effects** | **DF** | **AIC** |
| --- | --- | --- | --- | --- | --- | --- |
| Host seeking abundance | M1a | *An. arabiensis* | Time, Season, Livestock, Sat. deficit, Nets, House type, Distance Water | Village, HouseID , Date | 12 | 3999.8 |
|  | M1b | *An. arabiensis* | Season, Livestock, Sat. deficit, Nets, House type, Distance Water | Village, HouseID , Date | 11 | 3998.1 |
|  | M1c | *An. arabiensis* | Season, Livestock, Sat. deficit, House type, Distance Water | Village, HouseID , Date | 10 | 3997 |
|  | M1d | *An. arabiensis* | Season, Livestock, Sat. deficit, Distance Water | Village, HouseID , Date | 9 | 3996.9 |
|  | M1e | *An. arabiensis* | Livestock, Sat. deficit, Distance Water | Village, HouseID , Date | 8 | 3997.8 |
|  | **M1f** | ***An. arabiensis*** | **Livestock, Sat. deficit** | **Village, HouseID , Date** | **7** | **3997.3** |
| Host seeking abundance | M2a | *An. funestus* | Time, Season, Livestock, Sat. deficit, Nets, House type, Distance Water | Village, HouseID , Date | 12 | 3159.2 |
|  | M2b | *An. funestus* | Time, Season, Sat. deficit, Nets, House type, Distance Water | Village, HouseID , Date | 11 | 3157.2 |
|  | M2c | *An. funestus* | Time, Season, Sat. deficit, Nets, Distance Water | Village, HouseID , Date | 10 | 3155.5 |
|  | M2d | *An. funestus* | Time, Season, Sat. deficit, Distance Water | Village, HouseID , Date | 9 | 3154.3 |
|  | M2e | *An. funestus* | Time, Sat. deficit, Distance Water | Village, HouseID , Date | 8 | 3155.1 |
|  | **M2f** | ***An. funestus*** | **Time, Sat. deficit** | **Village, HouseID , Date** | **7** | **3155.7** |

**Supplementary Material 2:** The predicted impact of environmental variables on the estimated mean abundance of *An. arabiensis* and *An. funestus* host-seeking indoors from January 2012 to May 2015. Of the 7 variables tested for each vector species (timepoint, distance to water, saturation deficit, number of LLINs, house type, season, livestock presence), only those that had a statistically significant effect on their abundance are listed here. Chi-square values and associated p-values are as obtained by likelihood ratio test.

| **Species** | **Variable** | **Coefficient** | **χ2-value** | **p-value** |
| --- | --- | --- | --- | --- |
| *An. arabiensis* | Saturation deficit | -2.15 | 62.67 | <0.001 |
|  | Livestock present | -0.85 | 11.63 | <0.001 |
| *An. funestus* | Timepoint | -0.17 | 19.4 | <0.001 |
|  | Saturation deficit | -0.87 | 11.2 | <0.001 |

**Supplementary Material 3:** Model selection for indoor resting *An. arabiensis* and *An. funestus* *s.s*. with the chosen model marked in bold. Models were selected on the basis of significance of variables to the model fit, AIC-values and rule of parsimony as well as visual inspection of the plotted residuals against the fitted values. Time = Time point 1 to 14; Season = Wet/Dry; Livestock = Livestock owned (Yes/No); Sat. Deficit = Saturation Deficit of the air measured indoors; Distance Water = Estimated distance to closest breeding site; Nets = Number of LLINs used in household; House type = Building material house is mainly made of (mud/brick); Village = Village name (KID, LUP, MIN, SAG); Household = House ID; Date = Day/Month/Year of data collection.

| **Variable of interest** | **Name** | **Species** | **Fixed Effects** | **Random Effects** | **DF** | **AIC** |
| --- | --- | --- | --- | --- | --- | --- |
| Abundance resting indoors | M3a | *An. arabiensis* | Time, Season, Livestock, Sat. deficit, Nets, House type, Distance Water | Village, HouseID , Date | 12 | 2818.9 |
|  | M3b | *An. arabiensis* | Time, Season, Livestock, Sat. deficit, Nets, Distance Water | Village, HouseID , Date | 11 | 2816.9 |
|  | M3c | *An. arabiensis* | Time, Season, Livestock, Sat. deficit, Distance Water | Village, HouseID , Date | 10 | 2815.4 |
|  | M3d | *An. arabiensis* | Time, Season, Livestock, Distance Water | Village, HouseID , Date | 9 | 2816.7 |
|  | M3e | *An. arabiensis* | Time, Livestock, Distance Water | Village, HouseID , Date | 8 | 2816.8 |
|  | M3f | *An. arabiensis* | Time, Distance Water | Village, HouseID , Date | 7 | 2817.4 |
|  | **M3g** | ***An. arabiensis*** | **Time** | **Village, HouseID , Date** | **6** | **2818.1** |
| Abundance resting indoors | M4a | *An. funestus* | Time, Season, Livestock, Sat. deficit, Nets, House type, Distance Water | Village, HouseID , Date | 12 | 3472.9 |
|  | M4b | *An. funestus* | Time, Season, Livestock, Sat. deficit, House type, Distance Water | Village, HouseID , Date | 11 | 3471.5 |
|  | M4c | *An. funestus* | Time, Season, Livestock, Sat. deficit, Distance Water | Village, HouseID , Date | 10 | 3470 |
|  | **M4d** | ***An. funestus*** | **Time, Season, Livestock, Sat. deficit** | **Village, HouseID , Date** | **9** | **3469.1** |

**Supplementary Material 4:** The predicted impact of environmental variables on the estimated mean abundance of *An. arabiensis* and *An. funestus* *s.s.* resting indoors, outdoors and in animal sheds from January 2012 to May 2015. Of the 7 variables tested for each vector species (time point, distance to water, number of LLINs, house type, season, livestock presence, village), only those that had a statistically significant effect on their abundance are listed here. Chi-square values and associated p-values are as obtained by Wald- test.

| **Location** | **Species** | **Variable** | **Coefficient** | | **χ^2^- value** | **p-value** |
| --- | --- | --- | --- | --- | --- | --- |
| Indoors | *An. arabiensis* | Time | -0.13 | | 14.3 | <0.001 |
|  | *An. funestus* | Time | -0.06 | | 4.14 | 0.042 |
|  |  | Season | -0.54 | | 6.29 | 0.012 |
|  |  | Livestock | 0.48 | | 6.23 | 0.012 |
|  |  | Saturation Deficit | -1.01 | | 22.08 | <0.001 |
| Outdoors | *An. arabiensis* | Saturation Deficit | -0.41 | | 6.83 | 0.009 |
|  |  | Livestock | 0.55 | | 21.35 | <0.001 |
|  | *An. funestus* | Season | | -0.68 | 16 | <0.001 |
|  |  | Livestock | | 0.53 | 10.4 | 0.001 |
|  |  | Saturation Deficit | | -0.86 | 22.4 | <0.001 |
| Animal shed | *An. arabiensis* | Time | | -0.15 | 4.76 | 0.03 |
|  |  | Saturation Deficit | | -1.61 | 16.85 | <0.001 |

**Supplementary Material 5:** Model selection for outdoor resting *An. arabiensis* and *An. funestus* *s.s.* with the chosen model marked in bold. Models were selected on the basis of significance of variables to the model fit, AIC-values and rule of parsimony as well as visual inspection of the plotted residuals against the fitted values. Time = Time point 1 to 14; Season = Wet/Dry; Livestock = Livestock owned (Yes/No); Sat. Deficit = Saturation Deficit of the air measured indoors; Distance Water = Estimated distance to closest breeding site; Nets = Number of LLINs used in household; House type = Building material house is mainly made of (mud/brick); Village = Village name (KID, LUP, MIN, SAG); Household = House ID; Date = Day/Month/Year of data collection.

| **Variable of Interest** | **Name** | **Species** | **Fixed Effects** | **Random Effects** | **DF** | **AIC** |
| --- | --- | --- | --- | --- | --- | --- |
| Resting outdoors | M5a | *An. arabiensis* | Time, Season, Livestock, Sat. deficit, Nets, House type, Distance Water | Village, HouseID , Date | 12 | 12235.3 |
|  | M5b | *An. arabiensis* | Time, Season, Livestock, Sat. deficit, Nets, House type | Village, HouseID , Date | 11 | 12233.9 |
|  | M5c | *An. arabiensis* | Time, Livestock, Sat. deficit, Nets, House type | Village, HouseID , Date | 10 | 12232.8 |
|  | M5d | *An. arabiensis* | Time, Livestock, Sat. deficit, Nets | Village, HouseID , Date | 9 | 12232.8 |
|  | M5e | *An. arabiensis* | Time, Livestock, Sat. deficit | Village, HouseID , Date | 8 | 12232.2 |
|  | **M5f** | ***An. arabiensis*** | **Livestock, Sat. deficit** | **Village, HouseID , Date** | **7** | **12233.8** |
| Resting outdoors | M6a | *An. funestus* | Time, Season, Livestock, Sat. deficit, Nets, House type, Distance Water | Village, HouseID , Date | 12 | 5054.6 |
|  | M6b | *An. funestus* | Time, Season, Livestock, Sat. deficit, Nets, House type | Village, HouseID , Date | 11 | 5052.6 |
|  | M6c | *An. funestus* | Season, Livestock, Sat. deficit, Nets, House type | Village, HouseID , Date | 10 | 5051.4 |
|  | M6d | *An. funestus* | Season, Livestock, Sat. deficit, House type | Village, HouseID , Date | 9 | 5051.3 |
|  | **M6e** | ***An. funestus*** | **Season, Livestock, Sat. deficit** | **Village, HouseID , Date** | **8** | **5050.9** |

**Supplementary Material 6:** Model selection for *An. arabiensis* resting in animal sheds with the chosen model marked in bold. Models were selected on the basis of significance of variables to the model fit, AIC-values and rule of parsimony as well as visual inspection of the plotted residuals against the fitted values. Time = Time point 1 to 14; Season = Wet/Dry; Sat. Deficit = Saturation Deficit of the air measured indoors; Village = Village name (KID, LUP, MIN, SAG); Household = House ID; Date = Day/Month/Year of data collection.

| **Variable of Interest** | **Model Name** | **Species** | **Fixed Effects** | **Random Effects** | **DF** | **AIC** |
| --- | --- | --- | --- | --- | --- | --- |
| Abundance resting inside animal sheds | M7a | *An. arabiensis* | Time, Season, Sat. deficit | Village, HouseID , Date | 7 | 1610.6 |
|  | **M7b** | ***An. arabiensis*** | **Time, Sat. deficit** | **Village, HouseID , Date** | **6** | **1611.6** |

**Supplementary Material 7:**

Model selection for human blood index of *An. arabiensis* with the chosen model marked in bold. Models were selected on the basis of significance of variables to the model fit, AIC-values and rule of parsimony as well as visual inspection of the plotted residuals against the fitted values. Time = Time point 1 to 8; Season = Wet/Dry; Livestock = Livestock owned (Yes/No); Trap Type = Indoor trap (RH), Outdoor trap (RBU), Animal shed trap (RA); Village = Village name (LUP, MIN, SAG); Household = House ID; Date = Day/Month/Year of data collection.

| **Variable of Interest** | **Species** | **Name** | **Fixed effects** | **Random Effects** | **DF** | **AIC** |
| --- | --- | --- | --- | --- | --- | --- |
| Human Blood Index  overall | ***An. arabiensis*** | **M10a** | **Time, Season, Livestock, Trap type** | **Village, HouseID, Date** | **8** | **1371.1** |
| Human Blood Index  indoors | *An. arabiensis* | M11a | Time, Season, Livestock | Village, HouseID, Date | 6 | 469.9 |
|  | **An. arabiensis** | **M11b** | **Season, Livestock** | **Village, HouseID, Date** | **5** | **471.2** |
| Human Blood Index  outdoors | *An. arabiensis* | M12a | Time, Season, Livestock | Village, HouseID, Date | 6 | 852 |
|  | ***An. arabiensis*** | **M12b** | **Season, Livestock** | **Village, HouseID, Date** | **5** | **853** |

**Supplementary Material 8:** The predicted impact of environmental variables on the estimated mean Human Blood Index of *An. arabiensis* from January 2012 to May 2014. Of the 4 variables tested for each vector species (timepoint, season, livestock presence, trap type (Resting Bucket trap (RBU) or indoor back pack aspiration (RH)), only those that had a statistically significant effect on the Human Blood Index are listed here. Chi-square values and associated p-values are as obtained by likelihood ratio test.

| **Location** | **Species** | **Variable** | **Coefficient** | **χ^2^- value** | **p-value** |
| --- | --- | --- | --- | --- | --- |
| Overall | *An. arabiensis* | Time | -0.18 | 5.34 | 0.02 |
|  |  | Season | -1.5 | 12.66 | <0.001 |
|  |  | Livestock | -1.94 | 42.8 | <0.001 |
|  |  | Trap type (RBU or RH) | 1.11 | 42.8 | <0.001 |
| Indoors | *An. arabiensis* | Season | -2.07 | 6.24 | 0.012 |
|  |  | Livestock | -2.5 | 17.7 | <0.001 |
| Outdoors | *An. arabiensis* | Season | -1.4 | 8.66 | 0.003 |
|  |  | Livestock | -1.58 | 18.25 | 0.001 |

**Supplementary Material 9:**

Characterisation of the study population reporting the environmental conditions of the households sampled (table a). The environmental variables including season, saturation deficit, mean temperature and mean humidity, house type, number of nets per household, distance to the closest breeding site, and livestock presence, were tested for significant changes over time (table b). The monthly average rainfall in mm from 2012 to 2015 was checked for anomalies (c).

**SM 9a:**

| **Variable** | **2012** | **2013** | **2014** | **2015** |
| --- | --- | --- | --- | --- |
| Houses with livestock % | 37% | 16.3% | 79% | 37.5% |
| Houses made of mud % | 28% | 41% | 50% | 50% |
| Distance mean | 421m | 383m | 412m | 625m |
| Mean number of nets per household | 2.16 | 2.13 | 2.13 | 2.12 |

Table a): Characteristics of houses visited for data collection per year. Percentage of houses with livestock of the total number of houses visited, percentage of houses made of mud rather than brick, mean distance of houses from the closest breeding site and mean number of LLINs per household visited for data collection.

**SM 9b:**

| **Explanatory Variable** | | **Environmental response variables**  Predicted mean estimate (+/-95%CI) | | | | | |  |
| --- | --- | --- | --- | --- | --- | --- | --- | --- |
|  |  | Distance | Season | Housetype | Livestock | Nets | Sat. deficit |  |
| Timepoint | Coef | -0.001  (-0.02-0.018) | 0.05  (-0.4-0.5) | 0.24  (-0.5 - 1) | 16.85  (8.4-25) | -0.004  (-0.02 to -0.01) | 0.017  (-0.01-0.04) |  |
|  | p-value | 0.9 | 0.84 | 0.53 | <0.001* | 0.65 | 0.25 |  |

Table b): Predicted mean estimates (+/-95 CI) and p-values for the associations between environmental variables and time. Environmental variables: distance to closest breeding site (m), season (dry or wet), house type (mud or brick), livestock presence (yes or no), number of LLINs per house, saturation deficit indoors, as recorded at all houses where samples were collected at 14 time points from January 2012 to May 2015. Asterisks indicate a significant association between two variables.

**SM 9c:** Monthly rainfall data from the Climate Hazards group Infrared Precipitation with Stations (CHIRPS) dataset at 0.05º x 0.05º spatial resolution for the study period.

**
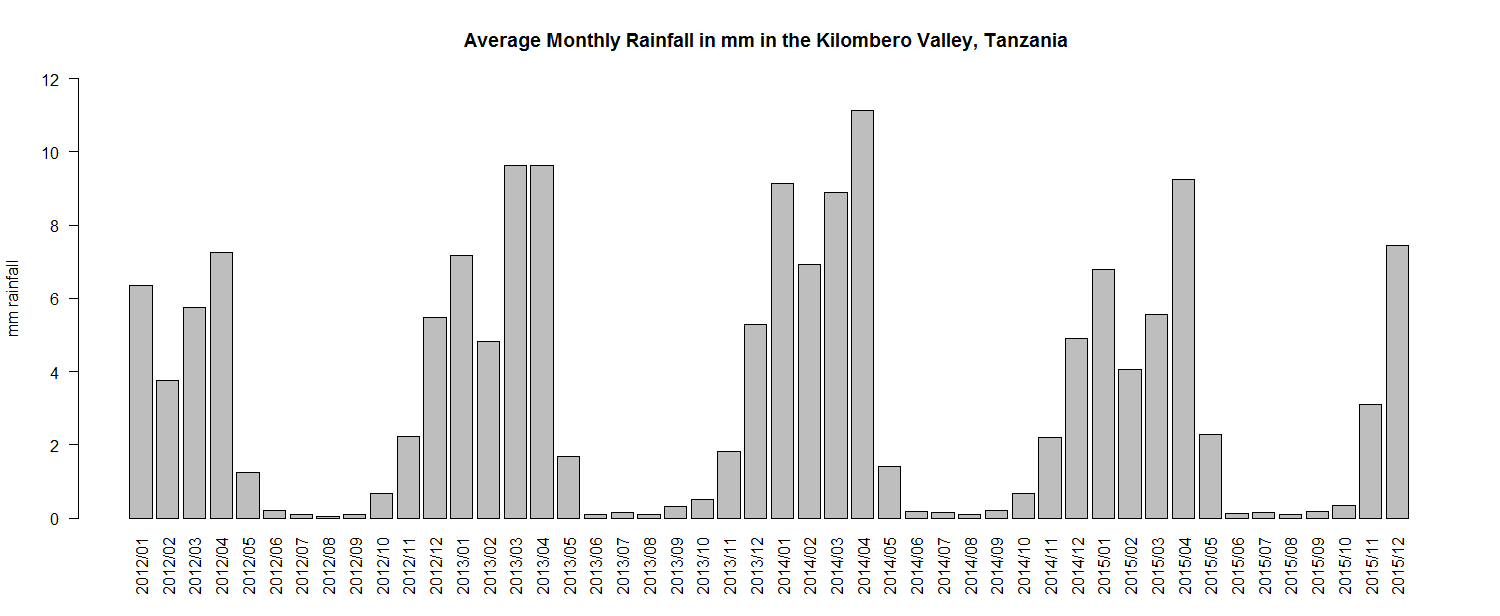
**

**Supplementary Material 10:** Experimental Design and classification of rainfall conditions during the study

Vector surveillance was carried out to test for evidence of long-term longitudinal shifts in the ecology (abundance and species composition) and behaviour (resting habitat and host choice) of malaria vectors between January 2012 and May 2015. Malaria vectors were repeatedly sampled in and around houses in each village in 3 to 4 consecutive day “blocks”. Villages were sampled sequentially with approximately 2 to 3 days between blocks to complete one round (e.g. one block in all villages). Two to three rounds of sampling were conducted each year to coincide with at least one wet and dry season, culminating in a total of 10 sampling rounds across ~3.5 years (SM1). A different group of houses was sampled during each block to maximize the spatial coverage of sampling within villages. Initially mosquitoes were sampled from 4 households per village in a block, which increased to 10 households in March 2013. In addition to the primary 10 rounds, ad hoc sampling was conducted at previously surveyed households to increase the sample size of recently fed mosquitoes as required for blood meal identification. In total, mosquitoes were collected from 350 households across 199 sampling days.

On the first day of each sampling block, an index house was selected on the basis of being accessible and the presence of residents to participate. Additional houses were recruited in the vicinity of the index house to achieve the required sample size (4 to 10 households per village), with spacing between individual houses no more than 100-200m.

| **%Year** | **Round** | **Rainfall condition** | **Village*** | **Date** | **Month** | **Houses** |
| --- | --- | --- | --- | --- | --- | --- |
| 2012 | 1 | wet | KID | 13th-17th | Feb | 4 |
| 2012 | 1 | wet | MIN | 26th -2nd | Feb-Mar | 4 |
| 2012 | 1 | wet | SAG | 7th-9th | Mar | 4 |
| 2012 | 1 | wet | LUP | 12th-16th | Mar | 4 |
| 2012 | 2 | wet | KID | 2nd-6th | Apr | 4 |
| 2012 | 2 | wet | MIN | 16th-20th | Apr | 4 |
| 2012 | 2 | wet | SAG | 7th-11th | May | 4 |
| 2012 | 2 | dry | LUP | 20th-24th | Aug | 4 |
| 2012 | 3 | dry | MIN | 3rd-7th | Sep | 4 |
| 2012 | 3 | dry | SAG | 10th-14th | Sep | 4 |
| 2012 | 3 | dry | KID | 17th-21st | Sep | 4 |
| 2012 | 3 | wet | LUP | 10th-14th | Dec | 4 |
| 2013 | Follow up^+^ | wet | LUP | 11th-15th | Feb | 10 |
| 2013 | Follow up^+^ | wet | LUP | 25th-1st | Feb-Mar | 5 |
| 2013 | Follow up^+^ | wet | LUP | 5th-7th | Mar | 5 |
| 2013 | Follow up^+^ | wet | SAG | 11th-15th | Mar | 10 |
| 2013 | Follow up^+^ | wet | SAG | 18th-22nd | Mar | 5 |
| 2013 | Follow up^+^ | wet | SAG | 25th-27th | Mar | 5 |
| 2013 | 4 | wet | SAG | 8th-12th | Apr | 10 |
| 2013 | 4 | wet | SAG | 15th-19th | Apr | 10 |
| 2013 | 4 | wet | MIN | 6th-10th | May | 10 |
| 2013 | 4 | wet | MIN | 13th-17th | May | 10 |
| 2013 | 4 | wet | LUP | 20th-24th | May | 10 |
| 2013 | 5 | wet | MIN | 27th-31st | May | 10 |
| 2013 | 5 | wet | LUP | 3rd-7th | Jun | 10 |
| 2013 | 5 | wet | LUP | 10th-14th | Jun | 10 |
| 2013 | 5 | dry | SAG | 17th-21st | Jun | 10 |
| 2013 | 6 | dry | KID | 8th-12th | Jul | 10 |
| 2013 | 6 | dry | MIN | 15th-19th | Jul | 10 |
| 2013 | 6 | dry | LUP | 22nd-26th | Jul | 10 |
| 2013 | 7 | wet | MIN | 21st-25th | Oct | 10 |
| 2013 | 7 | wet | KID | 18th-22nd | Nov | 10 |
| 2013 | 7 | wet | LUP | 25th-29th | Nov | 10 |
| 2013 | 7 | wet | SAG | 2nd-6th | Dec | 10 |
| 2013 | 7 | wet | MIN | 9th-13th | Dec | 10 |
| 2014 | 8 | wet | LUP | 13th-17th | Jan | 10 |
| 2014 | 8 | wet | SAG | 20th-24th | Jan | 10 |
| 2014 | 8 | wet | LUP | 11th-15th | Feb | 10 |
| 2014 | Follow up^+^ | wet | MIN | 18th-21st | Feb | 10 |
| 2014 | Follow up^+^ | wet | MIN | 3rd-7th | Mar | 10 |
| 2014 | 8 | wet | MIN | 17th-21st | Mar | 10 |
| 2014 | 8 | wet | KID | 24th-28th | Mar | 10 |
| 2014 | Follow up^+^ | wet | MIN | 2nd-4th | Apr | 5 |
| 2014 | Follow up^+^ | wet | MIN | 8th-11th | Apr | 5 |
| 2014 | 9 | dry | SAG | 30th-4th | Jun-Jul | 10 |
| 2014 | 9 | dry | MIN | 7th-11th | Jul | 10 |
| 2014 | 9 | dry | KID | 14th-18th | Jul | 10 |
| 2014 | 9 | dry | LUP | 21st-25th | Jul | 10 |
| 2014 | 10 | wet | MIN | 8th-12th | Dec | 10 |
| 2014 | 10 | wet | LUP | 15th-19th | Dec | 10 |
| 2015 | 10 | wet | SAG | 10th-24th | Apr | 10 |
| 2015 | 10 | wet | KID | 27th-1st | Apr-May | 10 |
| *Villages Kidugalo (KID); Lupiro (LUP); Minepa (MIN); Sagamaganga (SAG)  **^+^** Follow up sampling block for the collection of blood-fed resting mosquitoes | | | | | | |

**SM 10a:** Blocks of sample collection over the study period with corresponding round, date, location, and rainfall condition at the time and number of houses visited. Follow up sampling blocks for the collection of blood-fed resting mosquitoes are included in chronological order.

**SM 10b:** Method for classifying rainfall conditions during sampling

The start and length of the dry and wet seasons varied every year and were classified on the basis of model-derived estimates of mean rainfall for that particular month as compared to those over the whole study period (Jan 2011 to March 2015), with values above the threshold of 20% of the 5 year monthly average defined as “wet”; and those falling below as “dry”. Monthly rainfall data was obtained from two monitoring stations set up by the Kilombero Valley Teak Company – one in Mavimba (S08º25.2015’; E036º67.9735’), a village closely situated to Lupiro, Minepa and Sagamaganga and one in Kidugalo, the remaining village where sampling took place. Smoothed estimates of monthly rainfall were estimated for each month of study by fitting raw data to a harmonic seasonal linear regression model with eight sinusoidal covariates, four sine and four cosine with periods of 12, 6, 4 and 3 months (1). All eight covariates were significant (P < 0.05, R^2[adj] = 84.1%). Sampling blocks that fell within months in which the mean rainfall was above the threshold of 20% of the mean 5 year average were defined as “wet”; whereas those that fell below this threshold were defined as “dry”.

**Supplementary Material 11:** Information on datasets and number of samples used for the analysis of (1) resting and host-seeking mosquito abundance (2) Indoor and outdoor resting (3) Human blood index

|  |  | |  | |  | |  |  |  |  |
| --- | --- | --- | --- | --- | --- | --- | --- | --- | --- | --- |
| **Dataset** | | **Species** | **Villages** | **Houses** | **N^o^ of Complete Trapping Events** | | ***An. arabiensis*** | | ***An. funestus*** | |
| 1) Resting and Host-Seeking Abundance | | *An. arabiensis*  *An. funestus* | 4 | 350 | 500 | | 27141 | | 7051 | |
| 2) Indoor and Outdoor Resting | | *An. arabiensis*  *An. funestus* | 4 | 350 | 5070 | | 12745 | | 4341 | |
| 3) Human Blood Index^*^ | | *An. arabiensis*^+^ | 3 | 420 | 749 | | 2131 | | NA | |

* For Human Blood Index *An. arabiensis* mosquitoes from Kidugalo were excluded from the analysis due to insufficient sample size (n=9)

+ For the study on Human Blood Index only *An. arabiensis* mosquitoes were used
