# Supplementary material for: Emergence of behavioural avoidance strategies of malaria vectors in areas of high LLIN coverage in Tanzania
Source: Sci Rep. 2020 Sep 3;10:14527. doi: 10.1038/s41598-020-71187-4 (PMC7471940; doi:10.1038/s41598-020-71187-4)
Supplement: Supplementary file 4 — Supplementary Figure Legends [file 41598_2020_71187_MOESM4_ESM.docx]

Supplementary Material:

SM Figure 1:

Observed mean values with standard deviation of a) indoor host-seeking abundance of *An. arabiensis* and *An. funestus s.s*. collected from January 2012 to July 2015 and b) human blood index from *An. arabiensis* and *An. funestus* collected overall, indoors, outdoors and in animal sheds from January 2012 to July 2014.

SM Figure 2:

Observed mean abundance with standard deviation of resting *An. arabiensis* and *An. funestus* collected from January 2012 to July 2015 a) indoors b) in animal sheds and c) outdoors.
